# Computational evaluation of fluid flow influencing geometrical parameters of cyclone separator for Cement Kiln

**DOI:** 10.1371/journal.pone.0342112

**Published:** 2026-06-25

**Authors:** Mohnish Kumar, Harshad Deshpande, Sameer Bhosale, Himadri Majumder, Ch. Sateesh Kumar, Alemu Workie Kebede, Manish Deshmukh

**Affiliations:** 1 Department of Mechanical Engineering, PES’s Modern College of Engineering, Pune, Maharashtra, India; 2 Department of Mechanical Engineering, G H Raisoni College of Engineering & Management, Pune, Maharashtra, India; 3 Laboratory for Tribology and Interface Nanotechnology, Faculty of Mechanical Engineering, University of Ljubljana, Ljubljana, Slovenia; 4 Department of Mechanical Engineering, University of Coimbra, CEMMPRE, ARISE, Rua Luís Reis Santos, Coimbra, Portugal; 5 Department of Mechanical Engineering, Institute of Technology, Debre Markos University, Debre Markos, Ethiopia; 6 Department of Mechanical Engineering; AISSMS College of Engineering, Pune, Maharashtra, India; NED University of Engineering and Technology, PAKISTAN

## Abstract

Cyclone separators are integral to cement kiln operations, serving as key devices for mitigating air pollution and reducing particulate matter. Computational Fluid Dynamics (CFD) simulations enable visualization of internal flow distribution and provide quantitative insights into separator performance. A numerical CFD model was developed for the cyclone geometry, with the simulation results validated against existing experimental data. This study employs the design of experiments (DoE) technique to optimize design parameters and to examine how distinctive geometric features influence cyclone efficiency. An optimization study utilizing the design of experiments (DoE) technique was conducted to refine the design parameters for enhanced cyclone performance. This study investigated the impact of modifying key geometrical features of a cyclone separator on its performance metrics. The fluid flow performance influencing geometrical parameters considered in this study are the inlet height ratio (I_H_/D_b_ = 0.25 to 0.75), inlet width ratio (I_W_/D_b_ = 0.1 to 0.3), particle exit diameter ratio (D_e_/D_b_ = 0.2 to 0.4), bottom cone length ratio (L_c_/D_b_ = 1.75 to 2.75), and vortex finder diameter and length ratio (D_v_/D_b_ and L_v_/D_b_ = 0.25 to 0.75). Here, the term “ratio” refers to the ratio of the respective dimension to the cyclone barrel diameter (D_b_). The optimized design features an inlet height ratio of 0.5, an inlet width ratio of 0.21, a particle exit diameter ratio of 0.26, a bottom cone length of 2.31, and a vortex finder with a 0.5 in diameter and a 0.55 in length. This configuration resulted in a pressure drop of 285 Pa and an efficiency of 96%.

## 1 Introduction

Air pollution is a pressing environmental issue, with industrial emissions being a major source of particulate matter and other pollutants. To promote sustainable practices and protect public health, technologies such as electrostatic precipitators, Baghouse filters, Cyclone separators, and HEPA filters are indispensable. Cyclone separators, valued for their efficiency and reliability, are widely used for gas-solid and fluid-solid separations in industries such as cement kilns, food processing, and water treatment.

In cement kilns, cyclones are crucial for separating particulates from gases during production, using centrifugal force to achieve effective dust collection and heat recovery. This process is key to conserving energy and complying with environmental regulations. Cyclones also aid in preheating raw materials and reducing emissions, making cement manufacturing more sustainable and cost-efficient. Their simple yet dependable design ensures their essential role in modern systems, while Computational Fluid Dynamics (CFD) methods are used to enhance their design, addressing challenges such as turbulence and reducing pressure drop.

Recent studies have focused on innovative designs and optimization techniques to improve efficiency while minimizing pressure drop. Cortez et al. [1] analyzed cyclone designs, emphasizing the role of geometric adjustments in improving efficiency and reducing pressure drop. Pouria et al. [2] introduced a space-efficient cyclone design that achieved a 12.4% efficiency improvement over traditional models. Pandey et al. [3] used genetic algorithms and large-eddy simulations to optimize cyclone geometries, achieving up to a 43% reduction in pressure drop. Ravaliya et al. [4] critically examined empirical methodologies for evaluating cyclone separator efficiency in industrial settings, emphasizing operational conditions and standardized testing methods.

Toum et al. [5] used Industry 4.0 tools, such as machine learning, to monitor and analyze temperature and pressure in real time, preventing cyclone preheater blockages and improving safety and efficiency. Ravi et al. [6] evaluated cyclone separator designs, emphasizing the relationships among inlet velocity, pressure drop, and particle separation efficiency. Doe et al. [7] explored the design modifications in cyclone preheaters to enhance thermal efficiency and reduce energy consumption. Johnson et al. [8] investigate how geometric changes in cyclone separators affect dust collection and heat recovery in cement kilns. Lee et al. [9] used CFD to simulate and optimize cyclone separator designs for cement kiln applications. Wilson et al. [10] introduce energy-efficient cyclone models that reduce pressure drop while maintaining high separation efficiency. Taylor et al. [11] focused on innovative designs to address space constraints and improve operational efficiency in cement kilns. Harris et al. [12] examined the correlation between cyclone separator design parameters and overall kiln efficiency. Moore et al. [13] highlighted the use of multi-stage designs and refined geometries to improve cyclone performance in cement kilns. Wasilewski et al. [14] showed that varying the vortex finder diameter and insertion length in a square cyclone improved separation efficiency but increased the pressure drop. Elsayed et al. [15] compared the Stairmand design with a novel design and found that the novel design halved the pressure drop at the same flow rate. Elsayed et al. [16] also examined the effect of vortex finder dimensions using LES, discovering that smaller vortex finder diameters increased pressure drop and tangential velocity. Brar et al. [17] studied the effect of vortex finder position on cutoff diameter and pressure drop. Nassaj et al. [18] used CFD simulations to investigate how different inlet configurations affect flow behaviours. Their study suggests that strong vortex flows are crucial for efficient particle separation, and that the inlet design plays a significant role in generating them. Karagoz et al. [19] proposed a new cyclone design with improved dust-collection efficiency compared with older designs. Brar et al. [20] enhanced gas cyclone performance by adding multiple inlet ports, and Sun et al. [21] found that decreasing inlet height, width, and vortex finder diameter improved collection efficiency. Parvaz et al. [22] increased cyclone efficiency by enlarging the inner cone, thereby reducing the pressure drop. Wasilewski et al. [23] found that increasing the angle of the inlet duct bend improved separation efficiency. Izadi et al. [24] enhanced hydrocyclone efficiency by increasing the inlet flow rate. Elsayed et al. [25] studied the impact of inlet dimensions using the Reynolds stress model for turbulence, noting that larger inlet dimensions decreased pressure drop but also reduced tangential velocity. Babaoğlu et al. [26] analyzed the effect of inlet cross-section shape on static pressure and velocity, finding a maximum tangential velocity in rectangular inlets. Dziubak et al. [27] explored structural modifications to axial-flow cyclones to enhance separation efficiency. Dzmitry et al. [28] investigated how varying the inlet angle affected the performance of helical-roof inlet cyclones. Their findings indicated that inlet angles between 10° and 15° resulted in the best cyclone performance. Jadhav et al. [29] investigated the performance of flour mill cyclones at different flow rates. He compared the efficiency of a single-inlet cyclone with that of a symmetrical-inlet cyclone and found that the symmetrical-inlet cyclone performed better. Hsiao et al. [30] focused on improving the geometric configuration of cyclone separators, recommending specific ranges and ratios to enhance performance metrics. El-Emam et al. [31] explore the numerical methods employed in cyclone research and assess how several factors affect cyclone performance and flow behaviours in two-phase systems. Shahid et al. [32] performed a computational analysis of an industrial cyclone separator that initially exhibited low separation and collection efficiencies. Their study focused on the impact of varying inlet pressure and mass flow rate on the cyclone’s performance. The results showed that increasing tangential velocity improved separation efficiency. Zhang et al. [33] investigate the vortex flow field in a gas cyclone through the application of different vortex identification techniques. Their study deepens the understanding of vortex structures in gas cyclones, which are essential for improving cyclone performance, and contrasts the various methods used for vortex characterization. Using computational fluid dynamics, Dong et al. [34] explored the mechanisms and attributes of short-circuiting flow in gas cyclones. Our study supports their observations, showing that the main cause of short-circuiting is the differential timing of airflows entering the cyclone’s annular region. Areas susceptible to short-circuiting exhibit relatively short average particle residence times and considerable variability in retention durations. Parno et al. [35] examined the impact of various design changes on system efficiency. Their findings revealed that the inclusion of a dip leg significantly enhanced particle capture while concurrently minimizing pressure losses. The study by Dimitrijević et al. [36] focused on understanding the relationship between pressure drop and fluid flow in cyclones optimized for the separation of 1-micron particles. They examined empirical models of pressure loss to assess the variability of pressure drop across different cyclone geometries. Their research suggests that these predictions are sensitive to variations in cyclone dimensions and operating conditions. Feng et al. [37] conducted a CFD analysis to compare the flow behaviour of a novel cyclone separator with that of a conventional design. Their study comprehensively evaluated pressure drop and separation efficiency under varying operating conditions. The results indicated that the innovative separator achieved 45% higher separation efficiency for 1 μm particles than the traditional design, particularly at inlet velocities of 2–10 m/s. Notably, the novel separator demonstrated a 46% improvement in separation efficiency at an inlet flow rate of 2 m/s while maintaining a constant pressure drop. Zhang et al. [38] presented a novel cyclone separator design with an elliptical body, a potential enhancement over conventional circular designs. Assess the practical viability of this design, an experimental study was undertaken to compare the performance of circular and elliptical cyclone separators. The results revealed that elliptical cyclones showed modest improvements in separation efficiency (0.5-2%) and substantial reductions in pressure drop (10–30%) compared to their circular counterparts. These findings show that the advantages of the elliptical cyclone geometry, namely improved separation efficiency and decreased pressure drop, may also be beneficial in industrial-scale applications.

Although significant progress has been made in kiln optimization, a critical gap remains in the study of geometry-specific cyclone design for cement kilns. Most existing research emphasizes general operational parameters such as gas velocity and pressure drop while overlooking the influence of geometric configurations on system performance. This oversight limits the potential to maximize efficiency in dust collection, heat recovery, and emission control, all of which are essential for the sustainability of cement manufacturing.

To address this limitation, the present study applies a multi-factor optimization framework using the Design of Experiments (DoE) methodology with existing datasets. Prior studies [39,40] underscore the importance of optimizing operational variables but provide limited insight into geometry-driven design strategies. By integrating advanced computational approaches with DoE, this research aims to enhance the efficiency of cyclone separators and promote sustainable kiln operations. Unlike single-factor adjustments, which have shown only partial effectiveness, a multi-factor optimization approach offers the potential to improve separation efficiency and reduce pressure losses, thereby advancing both performance and environmental outcomes in cement kiln systems.

## 2 Numerical model

### 2.1 Multi-phase flow description and equations

Computational fluid dynamics (CFD) tools provide a virtual environment for creating solid models and simulating fluid behaviours within a flow domain. These tools offer numerous options for simulating fluid flow, considering both the design of the flow domain (e.g., cyclone dimensions and geometry) and operating conditions (e.g., material roughness, air density, and viscosity), as well as various turbulence models. Due to turbulence’s chaotic, unpredictable nature, analytically solving complex flow conditions is challenging. The Navier-Stokes equations, which describe fluid motion under dynamic conditions, are used to model these behaviours. Turbulence significantly affects these equations, influencing the characterization of flow conditions. Cyclone separators handle two-phase gas-solid flows. Flow gas stream models using incompressible and isothermal flow. The Navier-Stokes equations, averaged over the volume and in steady state, are used to solve for the gas phase.

In the case of incompressible and isothermal fluid flow, the Reynolds-Averaged Navier–Stokes (RANS) equations are employed to describe the mean flow field:

Continuity Equation (Mass Conservation):


$$\begin{array}{c} \frac{\partial \overset{―}{u_{i}}}{\partial x_{i}}=\text{0} \end{array}$$ (1)

This ensures conservation of mass, requiring that the net volumetric flux across any control volume is zero.

Momentum Equation (RANS Form):


$$\begin{array}{c} \frac{\partial (\rho \overset{―}{u_{i}})}{\partial x_{j}}=-\frac{\partial \overset{―}{\text{p}}}{\partial x_{i}}+\frac{\partial }{\partial x_{j}}\left[\mu \left(\frac{\partial \overset{―}{u_{i}}}{\partial x_{j}}+\frac{\partial \overset{―}{u_{j}}}{\partial x_{i}}\right)-\rho \overset{―}{{\overset{\prime }{\underset{\text{i}}{\text{u}}}\text{u}}_{\text{j}}}\right]+\rho f_{i} \end{array}$$ (2)

This represents momentum conservation, in which convective transport, pressure gradients, viscous stresses, turbulent stresses, and body forces are accounted for in the Reynolds stress term, as in turbulence modelling.

Turbulence Modelling
  Steady-State k–ε Model Equations

Turbulent kinetic energy (k):


$$\begin{array}{c} \frac{\partial }{\partial x_{i}}\left(\rho u_{i}k\right)=\frac{\partial }{\partial x_{j}}\left[\left(\mu +\frac{\mu_{t}}{\sigma_{k}}\right)\frac{\partial k}{\partial x_{j}}\right]+P_{k}-\rho \varepsilon \end{array}$$ (3)

Dissipation rate (ε):


$$\begin{array}{c} \frac{\partial }{\partial x_{i}}\left(\rho u_{i}\varepsilon \right)=\frac{\partial }{\partial x_{j}}\left[\left(\mu +\frac{\mu_{t}}{\sigma_{\varepsilon }}\right)\frac{\partial \varepsilon }{\partial x_{j}}\right]+C_{\text{1}\varepsilon }\frac{\varepsilon }{k}P_{k}-C_{\text{2}\varepsilon }\rho \frac{\varepsilon^{\text{2}}}{k} \end{array}$$ (4)

where:

$P_{k}$= production of turbulent kinetic energy

$\mu_{t}=\rho C_{\mu }\frac{k^{\text{2}}}{\varepsilon }$(eddy viscosity)

$C_{\mu },C_{\text{1}\varepsilon },C_{\text{2}\varepsilon },\sigma_{k},\sigma_{\varepsilon }$ are model constants

Steady-State k–ω Model Equations

Turbulent kinetic energy (k):


$$\begin{array}{c} \frac{\partial }{\partial x_{i}}\left(\rho u_{i}k\right)=\frac{\partial }{\partial x_{j}}\left[\left(\mu +\sigma_{k}\mu_{t}\right)\frac{\partial k}{\partial x_{j}}\right]+P_{k}-\beta^{*}\rho k\omega \end{array}$$ (5)

Specific dissipation rate (ω):


$$\begin{array}{c} \frac{\partial }{\partial x_{i}}\left(\rho u_{i}\omega \right)=\frac{\partial }{\partial x_{j}}\left[\left(\mu +\sigma_{\omega }\mu_{t}\right)\frac{\partial \omega }{\partial x_{j}}\right]+\alpha \frac{\omega }{k}P_{k}-\beta \rho \omega^{\text{2}} \end{array}$$ (6)

where:

$\mu_{t}=\rho \frac{k}{\omega }$ (eddy viscosity)

$\alpha ,\beta ,\beta^{*},\sigma_{k},\sigma_{\omega }$ are model constants

Steady-State RSM Model Equations

The decomposition of velocity into a mean and a fluctuating component introduces Reynolds stresses, which require closure. The Boussinesq hypothesis provides an approximation:

Reynolds Stress Closure:


$$\begin{array}{c} \rho \overset{―}{\overset{\prime }{\underset{\text{i}}{\text{u}}}\overset{\prime }{\underset{\text{j}}{\text{u}}}}=\mu_{t}\left(\frac{\partial \overset{―}{u_{i}}}{\partial x_{j}}+\frac{\partial \overset{―}{u_{j}}}{\partial x_{i}}-\frac{\text{2}}{\text{3}}\frac{\partial \overset{―}{u_{k}}}{\partial x_{k}}\delta_{ij}\right)-\frac{\text{2}}{\text{3}}\rho k\delta_{ij} \end{array}$$ (7)

This relates turbulent stresses to the mean strain rate via eddy viscosity, $\mu_{t}$, simplifying the governing equations but introducing modeling assumptions.

For cyclone separators, where swirling anisotropic turbulence dominates, the Reynolds Stress Model (RSM) is more appropriate:

Momentum Equation (RSM Form):


$$\begin{array}{c} \overset{―}{u_{j}}\frac{\partial \overset{―}{u_{i}}}{\partial x_{j}}=-\frac{\text{1}}{\rho }\frac{\partial \overset{―}{P}}{\partial x_{i}}+\nu \frac{\partial^{\text{2}}\overset{―}{u_{i}}}{\partial x_{i}\partial x_{j}}+\frac{\partial R_{ij}}{\partial x_{j}} \end{array}$$ (8)

Here, divergence of the Reynolds stress tensor $R_{ij}=\overset{―}{{\overset{\prime }{\underset{\text{i}}{\text{u}}}\text{u}}_{\text{j}}}$is explicitly included, allowing anisotropy and swirling structures to be captured.

Transport Equation for Reynolds Stress Tensor:


$$\begin{array}{c} \frac{\partial R_{ij}}{\partial t}+\overset{―}{u_{k}}\frac{\partial R_{ij}}{\partial x_{k}}=\frac{\partial }{\partial x_{k}}\left(\frac{\nu }{\sigma }\frac{\partial R_{ij}}{\partial x_{k}}\right)-\left(\frac{\partial \overset{―}{u_{j}}}{\partial x_{k}}R_{ik}+\frac{\partial \overset{―}{u_{i}}}{\partial x_{k}}R_{jk}\right)-C_{\text{1}}\frac{\varepsilon }{k}\left(R_{ij}-\frac{\text{2}}{\text{3}}\rho k\delta_{ij}\right)+C_{\text{2}}\left(P_{ij}-\frac{\text{1}}{\text{3}}P_{kk}\delta_{ij}\right)-\frac{\text{2}}{\text{3}}\delta_{ij}\varepsilon \end{array}$$ (9)

This equation governs the transport of Reynolds stresses, accounting for convection, diffusion, production, and dissipation. It provides a detailed description of turbulence dynamics essential for cyclone modelling.

Turbulence Production Term:


$$\begin{array}{c} P_{ij}=-{[R}_{ik}\frac{\partial \overset{―}{u_{j}}}{\partial x_{k}}+R_{jk}\frac{\partial \overset{―}{u_{i}}}{\partial x_{k}}] \end{array}$$ (10)

This term quantifies energy transfer from mean flow gradients into turbulent fluctuations, which is particularly significant in swirling cyclone flows.

Particle Tracking (Eulerian–Lagrangian Approach)

Cyclone separators rely on particle–gas interactions. The gas phase is modelled as a continuous Eulerian field, while particles are tracked individually using a Lagrangian framework with one-way coupling (the gas affects particles, but particles do not affect the gas flow due to their low concentration).

Particle Motion Equation:


$$\begin{array}{c} \frac{du_{pi}}{dt}=F_{d}\left(u_{i}-u_{pi}\right)+g_{i}\frac{\rho_{p}-\rho }{\rho_{p}} \end{array}$$ (11)

This describes particle acceleration due to drag and gravity, where $U_{pi}$ is particle velocity, $\rho_{p}$ particle density, and $F_{d}$ the drag force coefficient.

Drag Force Expression:


$$\begin{array}{c} F_{d}=\frac{\text{18}\mu C_{d}Re_{p}}{\rho_{p}\overset{\text{2}}{\underset{p}{D}}\cdot \text{24}} \end{array}$$ (12)

The drag force depends on fluid viscosity μ, particle diameter $D_{p}$, and drag coefficient $C_{d}$.

Particle Reynolds Number:


$$\begin{array}{c} Re_{p}=\frac{\rho_{p}D_{p}(u_{i}-u_{pi})}{\mu } \end{array}$$ (13)

This dimensionless number characterizes the relative importance of inertial and viscous forces acting on particles.

Cyclone Efficiency

The collection efficiency (η) is defined as the fraction of particles separated from the gas stream:


$$\begin{array}{c} \eta =\frac{m_{e}-m_{o}}{m_{e}}\times \text{100} \end{array}$$ (14)

Here, $m_{e}$ is the mass of particles introduced at the inlet, and $m_{o}$is the mass exiting with the clean gas. A restitution coefficient of 1 is assumed, implying perfectly elastic particle–wall collisions. This simplification focuses on translational motion, which dominates separation behavior.

### 2.2 Case description and Boundary conditions

To achieve computationally efficient simulations of cyclone separators, several simplifying assumptions are introduced. These include steady-state flow conditions, incompressible fluid behaviours, uniform particle properties, and the neglect of minor geometric irregularities.

Details of boundary conditions are given below:

I. Inlet and Outlet: The model typically stands for a simplified cyclone geometry with one inlet for the gas-particle mixture and one outlet for the clean gas.
II. Gas Inlet: The gas entering the cyclone is assumed to have a uniform velocity profile. This implies the gas speed is constant across the inlet area.
III. Clean Gas Outlet: The clean gas outlet maintains constant pressure, often set to zero gauge pressure (ambient pressure). Additionally, it’s assumed that there are no variations (gradients) in other flow properties, such as temperature or turbulence, at the outlet.
IV. Wall Conditions: The walls of the cyclone separator are modelled with no-slip boundary conditions. This implies that the gas velocity at the wall is zero, representing the friction between the gas and the solid surface.
V. Particle Trajectories: Uniform particles are injected at the inlet, and the number that escape from the gas outlet is used to calculate the cyclone efficiency.

Details of boundary conditions are given in Table 1.

**Table 1:** Boundary conditions.

Inlet	Outlet	Wall
Uniform Velocity inlet, 15 m/s	Pressure outlet, 0 Pa	No slip conditions
A particle size of 5 μm injected	Escape for particles	Rebound
500000 particles are injected	Escape for particles	Rebound

Although the flow inside cyclone separators is inherently turbulent and strongly swirling, steady-state Reynolds-averaged Navier–Stokes (RANS) simulations are widely used to predict global performance parameters, such as pressure drop and collection efficiency. The objective of the present study is to evaluate the comparative performance of different cyclone geometrical configurations based on mean flow characteristics rather than resolving instantaneous vortex fluctuations. Even though the turbulent, swirling flow inside a gas cyclone is complex, steady-state simulations were used to reduce computational cost relative to transient approaches. Moreover, cyclone separators typically operate under quasi-steady industrial conditions, where time-averaged quantities, such as pressure drop and separation performance, are of primary engineering interest. Therefore, the steady-state RANS framework adopted in this study is considered appropriate for the intended performance assessment and is consistent with similar CFD investigations reported in the literature [41,42]. In practice, cyclone systems operate under quasi-stationary conditions, allowing steady-state computational fluid dynamics (CFD) models to effectively represent mean flow behaviours, separation efficiency, and pressure losses. From an industrial perspective, the emphasis lies on long-term averages of efficiency and energy consumption rather than transient fluctuations, making steady-state RANS simulations a cost-effective and reliable approach for performance assessment. Since the present work employs steady-state simulations, the CFL number is not directly applicable. Numerical stability was ensured through suitable discretization schemes and convergence monitoring rather than explicit CFL control. The reported profiles are obtained from steady state simulations and therefore represent converged solutions rather than time-averaged data [43].

Pressure–velocity coupling was managed using the SIMPLE (Semi-Implicit Method for Pressure-Linked Equations) algorithm to ensure stable convergence. The spatial discretisation of the governing equations was performed using a second-order scheme [31, 33, 37] for both momentum and pressure to minimise numerical diffusion and improve accuracy. Turbulence equations were also discretized with second-order accuracy.

The pressure drop across the cyclone separator was evaluated as the difference between the area-averaged static pressures at the inlet and outlet boundaries. During steady-state convergence, the static pressure field was continuously monitored, and once the residuals and flow parameters had stabilized, the inlet and outlet static pressure values were recorded. The predicted pressure drop was calculated as the difference between the stabilised inlet and outlet pressures.

### 2.3 Numerical grid independence verification

Although hexahedral meshes are often preferred to reduce numerical diffusion, polyhedral meshes offer distinct advantages in complex geometries such as cyclone separators. Their flexibility and reduced cell count enable faster convergence while keeping comparable accuracy, making them particularly effective in CFD platforms [44]. A grid independence study confirmed that further refinement of the polyhedral mesh did not significantly alter key performance indicators, including pressure drop, thereby ensuring reliable predictions.

In this work, polyhedral grids were used, and grid independence was validated by plotting the pressure drop as a function of mesh density. Convergence was achieved when further refinement produced negligible changes in the results, with a mesh size of approximately 2.1 million elements sufficient to ensure grid-independent results. This confirms that the chosen polyhedral topology offers both computational efficiency and accuracy in predicting cyclone separator performance.

**Fig 1:**
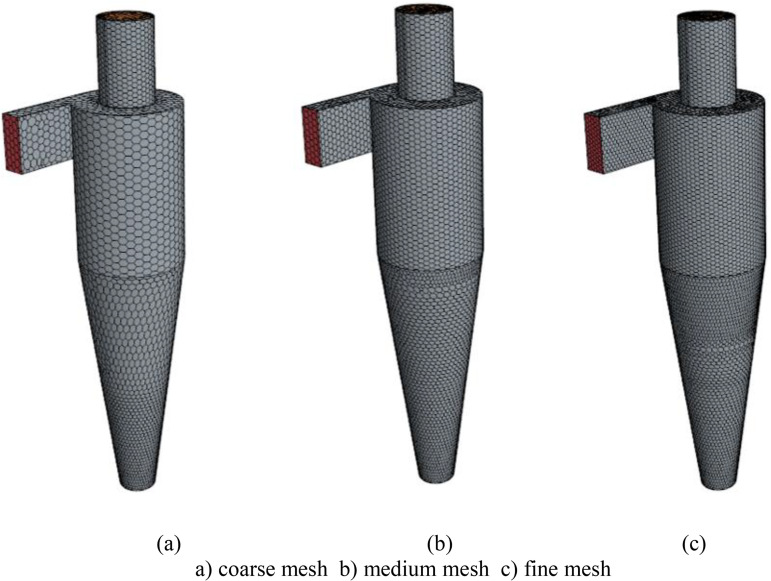
Mesh details.

Mesh density is crucial for accurately capturing flow characteristics. Therefore, we conducted a mesh sensitivity study using five different mesh sizes ranging from 0.5 to 4.5 million elements. Fig 1 illustrates three representative meshes: Fig 1(a), coarse, coarsest (0.5-1.5 million), Fig 1(b), medium (1.5-2.5 million), and Fig 1(c), fine, finest (3 - 4.5 million). CFD simulations were used to predict static pressure drops for each mesh density. Table 2 presents a comparison of the CFD-predicted static pressure drops.

**Table 2:** Pressure drops vs number of cells.

Grid Type	Number of cells	Static Pressure Drops (Pa)
Coarsest	0.5 million	354
Coarse	1.1 million	339.6
Medium	2.3 million	311
Fine	3.0 million	308.8
Finest	4.5 million	308.7

The difference between the coarse and fine mesh predictions was 12.9%, while the difference between the medium and fine mesh predictions was only 0.71%. As shown in Table 2, the fine mesh had a deviation of less than 0.71% compared to the medium mesh. However, the fine mesh required 150% more computational time. Therefore, the medium mesh with 2.3 million elements was chosen for further analysis.

### 2.4 Selection of turbulence models on the swirling flow field in a cyclone separator

The swirling flow inside a cyclone separator generates substantial turbulence, making precise modelling of these turbulent phenomena essential for predicting the flow field and investigating the influence of turbulence on flow and particle behaviours. Three turbulence models were employed: the k-ε model, the SST k-ω model with curvature correction, and the RSM model. The SST k-ω model is particularly well-suited for swirling flows, as it solves two transport equations for turbulence kinetic energy (k) and the specific dissipation rate (ω). For simpler cases, the SST k-ω model with curvature correction is often sufficient, as it is designed for curvature-dominated flows and is computationally more efficient than RSM.

**Fig 2:**
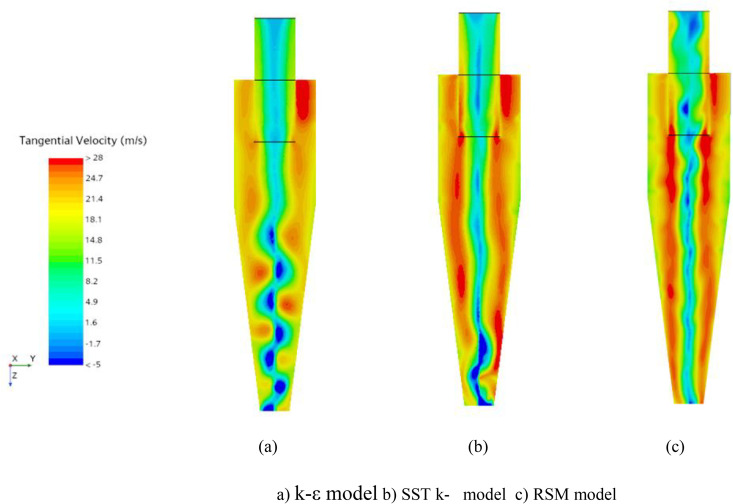
Tangential velocity for three different turbulence models.

The RSM model Fig 2(c), which incorporates seven additional transport equations to capture turbulence induced by shear stress, shows superior accuracy in modelling the complex swirling flow within the cyclone separator. Although computationally more demanding, the RSM model is the preferred turbulence model for simulating cyclonic flow. Tangential and axial velocities are key parameters for evaluating cyclone performance. The tangential velocity, as the dominant component, generates the centrifugal force that is essential for particle separation. The swirling motion generates centrifugal force, causing particles to separate from the gas stream. Fig 2 shows tangential velocity contours for the three turbulence models. Near the wall, both the k-ε model in Fig 2 (a) and the SST k-ω model in Fig 2 (b) predict higher tangential velocities than RSM. While the tangential velocity distributions are comparable in the inner region, significant differences arise near the wall. These variations might be attributed to differences in the three turbulence models’ ability to capture shear fluctuations in turbulence near the wall.

Three turbulence models were evaluated: the standard k−ε, the SST k−ω with curvature correction, and the Reynolds Stress Model (RSM). Among these, the RSM demonstrated superior accuracy in being anisotropic turbulence and complex swirling structures characteristic of cyclone separators, despite its higher computational demand. This justifies its selection as the primary turbulence model for the present study.

Appropriate wall treatments were applied for each model to ensure consistency in near-wall predictions: standard wall functions for the k−ε and SST k−ω models, and enhanced wall treatment for the RSM. In addition, the non-dimensional wall distance ($Y^{+}$) was carefully monitored to ensure the validity of the applied wall functions. For the RSM with enhanced wall treatment, $Y^{+}$ values were kept within the recommended range (typically 30–300 for wall-function approaches), ensuring accurate resolution of near-wall turbulence effects without high computational cost. The combination of RSM with enhanced wall functions and controlled $Y^{+}$ values provided a reliable representation of the flow field, particularly in regions dominated by strong swirl and turbulence anisotropy.

### 2.5 Solution stabilization and convergence assessment

To ensure accurate resolution of the governing conservation equations in CFD, residual thresholds for continuity and momentum are typically set between 10^-3 and 10^-4. Within these limits, the flow field is numerically stable, enabling reliable prediction of performance metrics such as pressure drop and separation efficiency. For one-way Discrete Phase Model (DPM) simulations, particle trajectories show reduced sensitivity to residual variations, and convergence is generally attained under the same residual criteria. In line with the recommendations of Kumar et al. [45], convergence verification extends beyond residual monitoring to include physical parameters such as pressure gradients and other system-specific indicators, ensuring both numerical accuracy and physical consistency in complex geometrical domains.

To assess iterative convergence, simulations were performed with three different residuals: 10−3, 10−4, and 10−6, under the same computational settings. The simulated pressure drop values were obtained with residual thresholds of ${\text{10}}^{-\text{3}}$, ${\text{10}}^{-\text{4}}$, and ${\text{10}}^{-\text{6}}$ were nearly identical. The deviation between the result (pressure drop) obtained for the (least stringent criterion) residuals level 10^−3^ and 10^−6^ (the most stringent) was 0.23%. Whereas the magnitude of deviation was 0.22%. for the pressure drop attained with residual levels 10^−4^ and 10^−6^ (the most stringent), which lies within the numerical uncertainty of the solver shown in Table 3. However, it was observed that lowering the residual threshold to 10^−6^, the computational time increased by approximately 10%. These results confirm that adopting a residual criterion of 10^−4^ is sufficient to ensure iterative convergence while maintaining accuracy and reducing computational cost.

**Table 3:** Influence of Residual Threshold on Pressure Drop Prediction.

Residuals level	Pressure drops (Pa)	% deviation from $10^{-6}$
${\text{1}\text{0}}^{-\text{3}}$	284.97	0.22
${\text{1}\text{0}}^{-\text{4}}$	285.00	0.23
${\text{1}\text{0}}^{-\text{6}}$	285.63	--

### 2.6 Validation of the present CFD model

To verify the accuracy of the present CFD model results, the predictions must be compared with experimental observations. This was assessed by comparing the predicted pressure drop, tangential velocity, and axial velocity distributions with the experimental results reported by Hoekstra [46].

**Fig 3:**
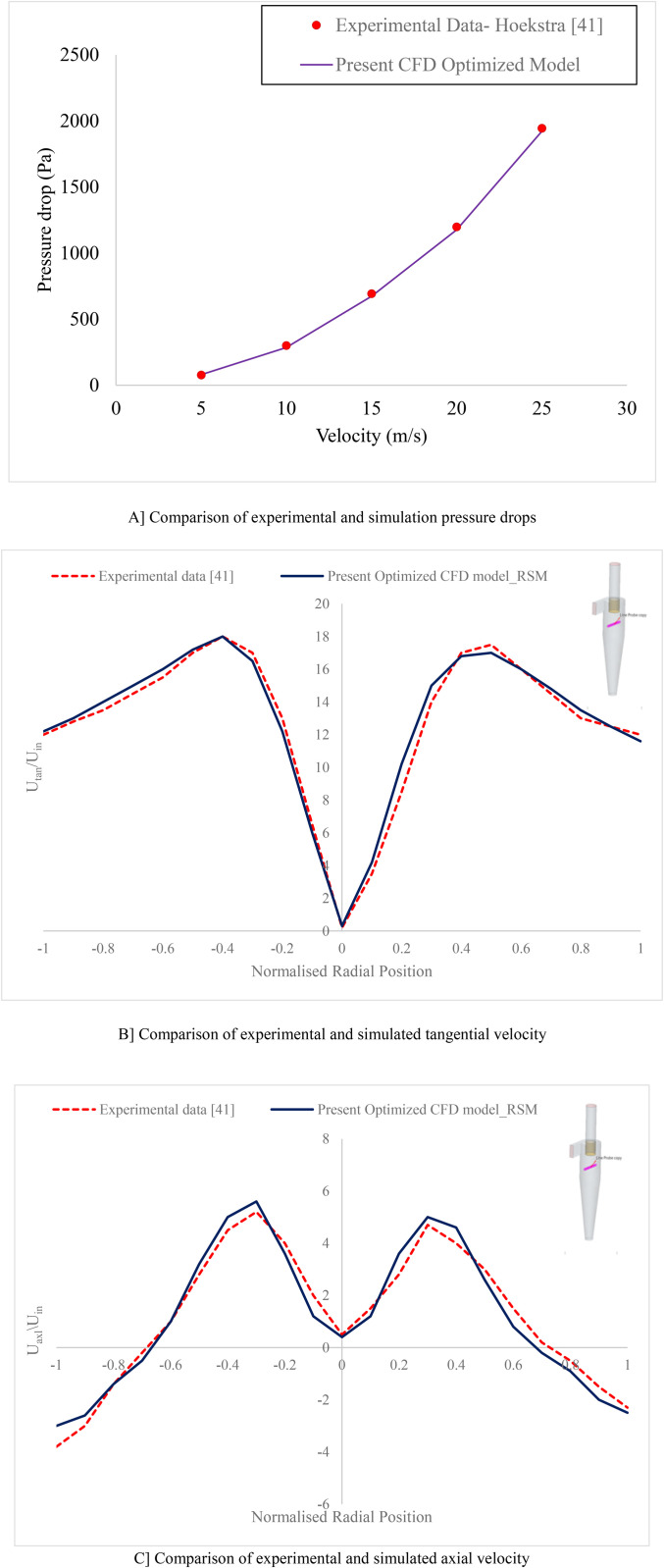
Validation of present CFD model.

The pressure drop of the optimized cyclone has been analyzed for five different velocities ranging from 5 to 25 m/s. The predicted pressure drops for the optimized cyclone design (present work) were compared with experimental data of Hoekstra [46], showing agreement within 5%. The pressure distribution, illustrated in Fig 3A, shows that the predicted pressure drop closely matches experimental results. At 10 m/s, the optimized design exhibits a pressure drop of 285 Pa, compared to the Hoekstra [46] experimental value of 300 Pa. While some discrepancy is expected due to inherent modelling uncertainties, the consistent improvement relative to Hoekstra’s cyclone geometry.

The difference between the inlet and outlet pressures can be used to calculate the cyclone’s experimental pressure drop [47]. Estimating the total pressure drop (static pressure plus dynamic pressure) is more accurate because it accounts for the variation in kinetic energy of the flow between the inlet and outlet sections. When the static and total pressure drops from the experimental data [46] are compared, the CFD prediction (present model) shows a very slight discrepancy between the estimated and observed values. An order-of-magnitude error of 5% between the predicted and experimental pressure drop was observed under identical simulation settings and is therefore attributed to the geometric modification rather than numerical uncertainty. The value of the error of order of 5% or below is the same as the experimental error [47].

The predicted axial and tangential velocity distributions were validated against Hoekstra [46] Laser Doppler Anemometry LDA measurements obtained along a radial line at (r/R = −1 to +1) and plane at 0.75 times cyclone diameter (z/D_b_ = 0.75) inside the cylindrical section of a Stairmand high-efficiency cyclone separator, as illustrated in Fig 3B, 3C. In view of the comparison, the magnitude of mean axial (U_axl_) as well as tangential (U_tan_) velocities at a distance of 0.75D from the cyclone top are normalized by dividing the value of the inlet velocity (U_in_). The velocity distribution obtained with the present RSM CFD model simulation matches the experimental velocity profile. The comparison shows satisfactory agreement with experimental observations, particularly in capturing the vortex core region and near-wall velocity gradients, demonstrating the reliability of the present numerical methodology for cyclone flow. The accuracy of the CFD model used in this work is confirmed by the satisfactory agreement between the two parameters, namely tangential and axial velocities, obtained from the two different techniques, as shown in Fig 3B and 3C.

The pressure drop and cyclone efficiency values from the experimental data [46] are 300 Pa and 85%, respectively. It was revealed that the simulated pressure drop (present optimized design) was 285 Pa, and the cyclone efficiency of 96%. The present optimized design of a cyclone separator effectively balances pressure drop and efficiency, indicating a well-performing cyclone design.

Nevertheless, the overall agreement confirms that the Reynolds Stress Model (RSM) used in the present CFD work provides a reliable representation of the highly anisotropic swirling flow within the cyclone and can predict separator performance with reasonable accuracy. This supports the conclusion that the optimized design provides a measurable improvement in hydraulic performance within the accuracy limits of the present CFD methodology and can contribute to long-term energy savings.

## 3 Optimization of design parameters through doe

Cyclone separators are widely used in cement kilns for separating particles from gas streams. A key challenge in cyclone design is to balance high particle capture efficiency (i.e., the percentage of particles captured) with minimal energy consumption (i.e., a low pressure drop within the cyclone). This study focuses on improving the design of cyclones typically used in cement plant grinding mills. These cyclones, typically ranging from 0.5 to 1.5 meters in diameter, play a crucial role in separating finer particles from the air stream after the grinding process (grinding clinker). In this application, a cyclone diameter of 1m was considered as a reference for the optimization process. By optimizing the design of these cyclones, engineers can improve their performance and efficiency in capturing fine particles while minimizing the energy required for operation. This translates to cost savings and environmental benefits for cement plants. Other dimensions concerning the cyclone body diameter are presented in Table 4.

**Table 4:** Present model- non-dimensional parameters.

Sr. No.	Parameters	Range
1	Inlet height ratio (**I**_**H**_**/D**_**b**_)	0.25 to 0.75
2	Inlet width ratio (**I**_**W**_**/D**_**b**_)	0.1 to 0.3
3	Particle exit diameter ratio **(D**_**e**_**/D**_**b**_)	0.2 to 0.4
4	Bottom cone length ratio **(L**_**c**_**/D**_**b**_)	1.75 to 2.75
5	Vortex diameter ratio **(D**_**v**_**/D**_**b**_)	0.25 to 0.75
6	Vortex length ratio **(L**_**v**_**/D**_**b**_)	0.25 to 0.75

**Fig 4:**
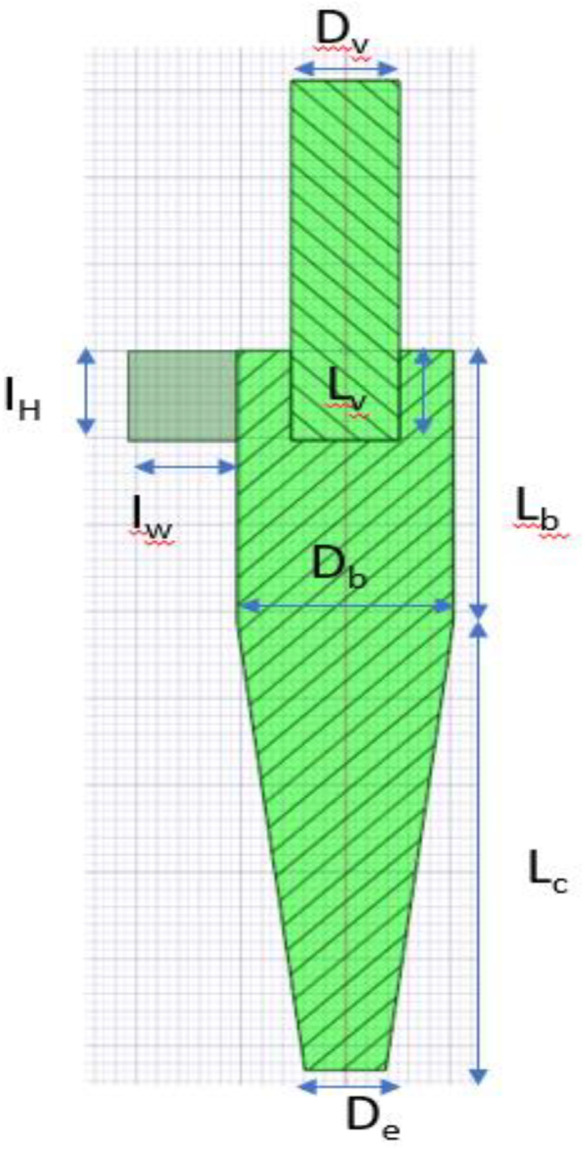
Cyclone layout.

A preliminary investigation was conducted to select the ideal mesh type and turbulence model for the simulations before optimizing cyclones using DOE. A medium-density mesh and the Reynolds Stress Model (RSM) for turbulence modelling are used in the configuration selected for this numerical analysis. Design optimization using DOE techniques is implemented across four different scenarios. The parameters that are considered are shown in Fig 4.

Design of Experiments (DOE) is a powerful technique for optimizing the design parameters of cyclone separators. It enables a methodical examination of various design scenarios to achieve optimal performance in pressure drop and collection efficiency.

### 3.1 Scenario 1: Inlet dimensions optimization

In the first scenario, the focus is on varying the inlet dimensions, specifically the height and width. The inlet height ratio (I_H_/D_b_) ranges from 0.25 to 0.75, and the width ratio (I_W_/D_b_) ranges from 0.1 to 0.3. To determine the optimal inlet dimensions that maximize separation efficiency while minimizing pressure loss, a 30 DOE experiment was carried out using various combinations.

### 3.2 Scenario 2: Particle exit diameter and cone height optimization

The second scenario explores the impact of changing the particle exit diameter ratio (bottom cone) from 0.2 to 0.4 and the cone height ratio from 1.75 to 2.75 to maximize overall performance.

### 3.3 Scenario 3: Vortex finder optimization

In the third scenario, the vortex finder length and diameter are varied, each ranging from 0.25 to 0.75. Another series of 30 DOE experiments was conducted to identify the optimal configuration that ensures high efficiency and a low pressure drop.

### 3.4 Scenario 4: Comprehensive parameter optimization

The final scenario involves a comprehensive approach in which six parameters were varied: inlet height, inlet width, particle exit diameter, cone leg, vortex height, and vortex diameter. This extensive DOE analysis aims to optimize the cyclone separator’s performance by examining the interplay among these critical factors.

By systematically varying these parameters and analyzing the results using DOE, we can achieve an informed and optimized design for the cyclone separator. This approach ensures the separator works at peak efficiency with minimal pressure drop, leading to improved overall performance.

## 4 Results and discussion

This section integrates the simulation results, focusing on three critical flow characteristics—pressure drop, axial velocity distribution, and tangential velocity distribution. By correlating these with efficiency metrics. The study highlights the key geometric factors that govern particle separation and energy consumption. The six geometric parameters, as per Table 4, were examined systematically. The optimization was conducted at the design stage using coupled nonlinear objective functions within the Design of Experiments (DoE) framework. The nonlinear nature of these objective functions, combined with geometric constraints, underscores the complexity of the optimization process but demonstrates the potential of multi-factor DoE methods to achieve reliable and efficient cyclone designs.

### 4.1 Influence of Inlet dimensions

Need for a multi-objective optimization approach to decide the ideal inlet dimensions for a cyclone separator. This is because two crucial factors need to be balanced: collection efficiency and pressure drop. The ranges of the inlet height and width vary during DOE, as shown in Table 5. A total of 30 DOE is performed.

**Table 5:** Variation of inlet parameters.

Sr. No.	Parameters	Range
1	Inlet height ratio (**I**_**H**_**/D**_**b**_)	0.25 to 0.75
2	inlet width ratio (**I**_**W**_**/D**_**b**_)	0.1 to 0.3

**Fig 5:**
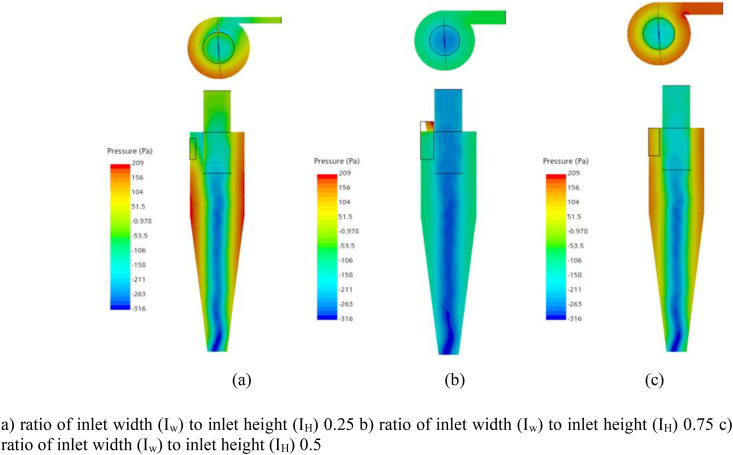
Pressure contours for three inlet designs.

Inlet width and height have opposing effects on pressure drop and cyclone efficiency. A wider inlet reduces pressure drop, saving energy, but at the cost of lower collection efficiency, allowing larger particles to pass through (increased cut-off diameter). The pressure drop here decreases with increasing inlet dimensions, as shown in Fig 5.

Fig 5(b) shows the higher inlet dimension; hence, the pressure drop is low. This is intuitive, as a larger opening offers less resistance to the flow and reduces the strength of the swirling vortex. In the other two designs, due to the high swirling velocity, a region of lower pressure (negative-pressure zone) exists in the main area (also known as the forced-vortex zone). The most significant pressure variations occur radially (outward from the centre). The design shown in Fig 5(a) has the highest average static pressure, exceeding those in Fig 5(b) (lowest) and 5(c) by 4.8% and 1.5%, respectively. Therefore, the design shown in Fig 5(b) is likely to experience the greatest pressure drop.

**Fig 6:**
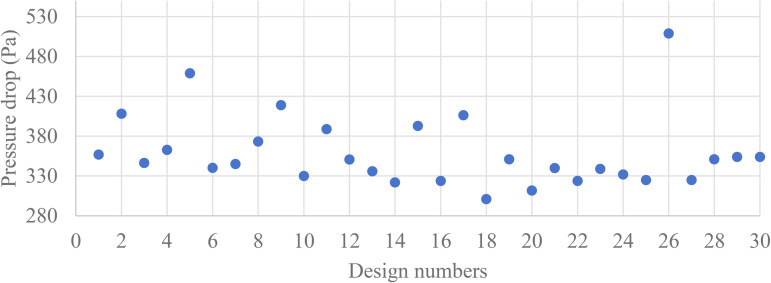
Pressure drop for all designs.

The overall change in pressure for 30 DOE designs is given in Fig 6. From the graph, it is clear that the pressure drop is slower in design 18, which corresponds to an inlet width (I_w_) to inlet height (I_H_) of 0.75.

**Fig 7:**
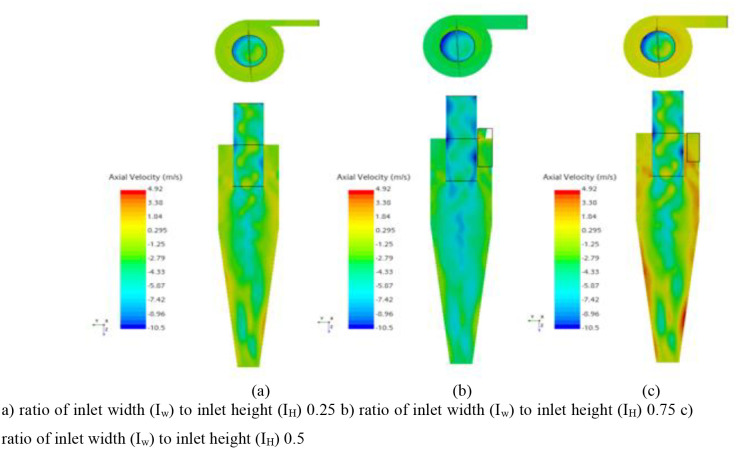
Axial velocity contours for three inlet designs.

The axial velocity is related to the downward and upward flow streams. Axial velocity is the part of fluid velocity directed along the longitudinal axis of a cyclone separator. The axial velocity profiles differ slightly between designs, as shown in Fig 7.

A wider inlet allows some incoming flow to directly affect the vortex finder, bypassing the swirling motion crucial for separation. Changes in inlet width or height have minimal influence on axial velocity, except near the central region, as shown in Fig 7. Fig 7 (a) shows a cyclone with a smaller inlet dimension, while Fig 7 (b) depicts an overdesigned cyclone with the largest inlet dimension. Fig 7 (c) presents the optimized design of the inlet dimension ratio.

**Fig 8:**
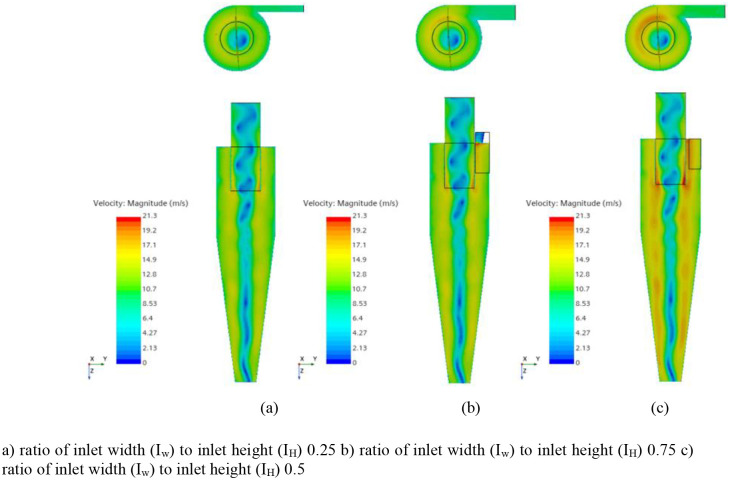
Velocity contours for three inlet designs.

Fig 8 shows the velocity magnitude for three different inlet dimensions. For three different inlet dimensions, the magnitude of the flow velocity entering the cyclone should be comparable. Once inside the cyclone, the rate of change of velocity should be minimal, resulting in a relatively uniform velocity distribution and minimal variations in axial velocity along the central axis. Fig 8 (a) shows a cyclone with a smaller inlet dimension, while Fig 8 (b) depicts an overdesigned cyclone with the largest inlet dimension. Fig 8 (c) presents the optimized inlet dimension ratio.

**Fig 9:**
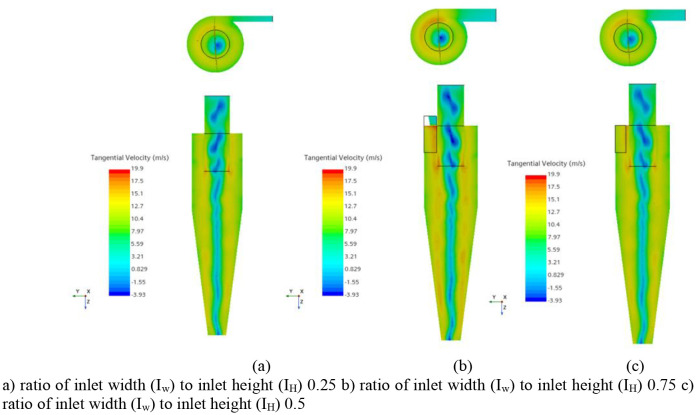
Tangential velocity contours for three inlet designs.

Fig 9 illustrates that the average tangential velocity rises significantly as you move outwards from the centre of the cyclone. It reaches a maximum value at a specific radius and then decreases gradually towards the wall.

A larger inlet area, Fig 9 (b), leads to lower tangential velocity due to the increased cross-sectional area for flow. This can reduce the centrifugal force, potentially affecting particle separation efficiency. As fluid enters the cyclone, it gains angular momentum. Reducing the inlet area forces the fluid to accelerate to conserve momentum, resulting in a higher tangential velocity, as illustrated in Fig 9(a). Improving the inlet dimension, as shown in Fig 9(c), requires balancing the advantages of increased tangential velocity and particle-separation efficiency against potential drawbacks such as higher pressure drop and increased energy consumption.

**Fig 10:**
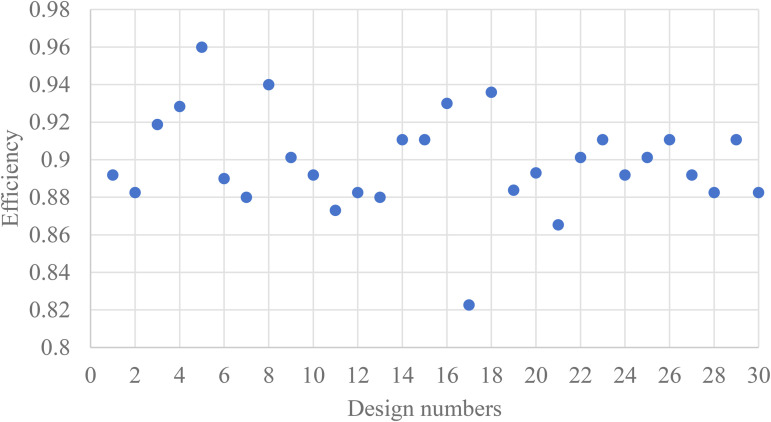
Cyclone efficiency for all designs.

Cyclone efficiency for all 30 designs is shown in Fig 10. Design 5 is more efficient but entails a higher pressure drop. Design 5 corresponds to an inlet width (Iw) to inlet height (IH) ratio of 0.36.

An optimal inlet width-to-height ratio of 0.42 in cyclone separators effectively balances pressure drop and separation efficiency. This configuration yields a pressure drop of 324 Pa and an efficiency of 0.93, showing a well-performing design (design 16). This balance is crucial for designing energy-efficient and effective particle separation systems.

### 4.2 Influence of bottom cone diameter (particle exit)

The ranges of the inlet height and width vary during the design of the experiment (DOE), as shown in Table 6. A total of 30 DOEs were performed.

**Table 6:** Variation of bottom cone parameters.

Sr. No.	Parameters	Range
1	Particle exit diameter ratio (**D**_**e**_**/D**_**b**_)	0.2 to 0.4
2	Cone length ratio (**L**_**c**_**/D**_**b**_)	1.75 to 2.75

**Fig 11:**
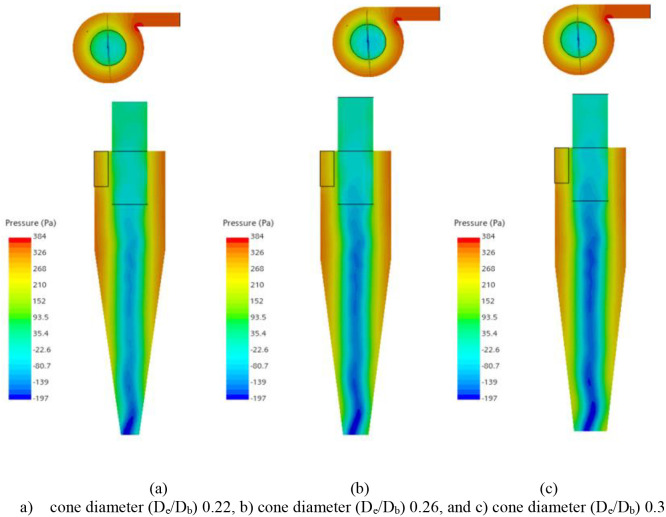
Pressure contours for three bottom cone designs.

A larger cone diameter generally results in a lower pressure drop due to the increased cross-sectional area, which reduces the flow resistance as shown in Fig 11(c).

Conversely, a smaller cone diameter increases the pressure drop as the fluid must pass through a more confined space, leading to higher frictional losses as shown in Fig 11(a). In Fig 11(b), a cone diameter of 0.26D achieves the best balance of pressure drop, resulting in efficient cyclone operation.

**Fig 12:**
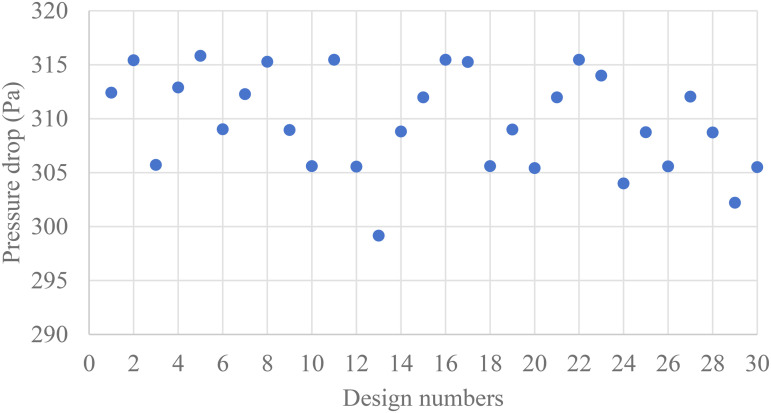
Pressure drop Vs designs.

Pressure drops for all 30 DOE are illustrated in Fig 12. The lowest pressure is observed in design 13, with a cone diameter of (D_e_/D_b_) 0.26. The pressure drop across a cyclone is directly influenced by the size of its bottom cone opening. A wider opening typically results in lower resistance to particle flow, thereby reducing the pressure drop. Conversely, a narrower opening can increase the resistance, leading to a higher pressure drop.

**Fig 13:**
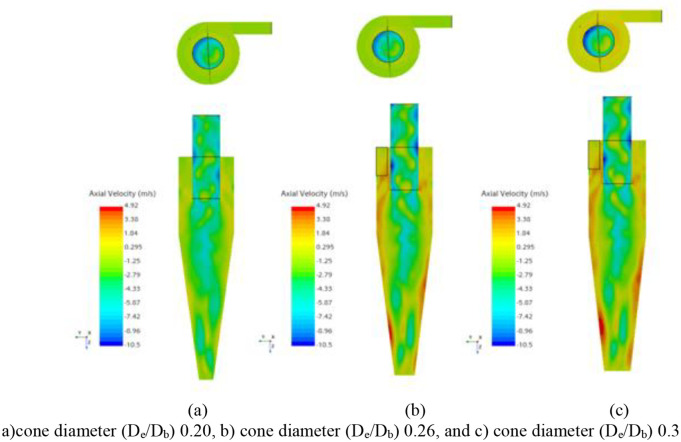
Axial velocity contours for three bottom cone designs.

Axial velocity refers to the part of the fluid velocity parallel to the cyclone’s central axis. The cone diameter influences the axial velocity profile within the cyclone, given in Fig 13. The bottom cone diameter of a cyclone directly affects its axial velocity. A larger diameter generally leads to higher axial velocity, while a smaller diameter results in lower axial velocity, as shown in Fig 13(a) and Fig 13(c), respectively. In Fig 13(b), a cone diameter of 0.26D provides an optimal balance of axial velocity, leading to efficient cyclone operation.

**Fig 14:**
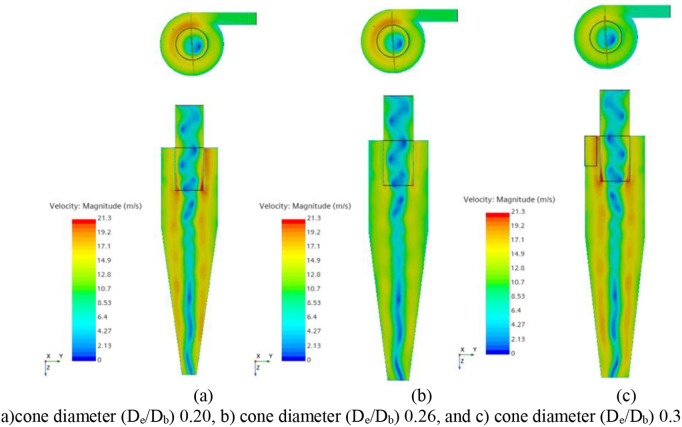
Velocity contours for three bottom cone designs.

Velocity contours are shown in Fig 14. Excessive velocities can cause particle re-entrainment and reduce separation efficiency. Conversely, a larger cone diameter tends to decrease axial velocity, which might help avoid re-entrainment but could also reduce the effectiveness of particle separation. Fig 14(a) shows a cyclone with a smaller cone diameter, Fig 14(c) shows a cyclone with the largest cone diameter, and Fig 14(b) presents the optimized cone diameter of 0.26D.

**Fig 15:**
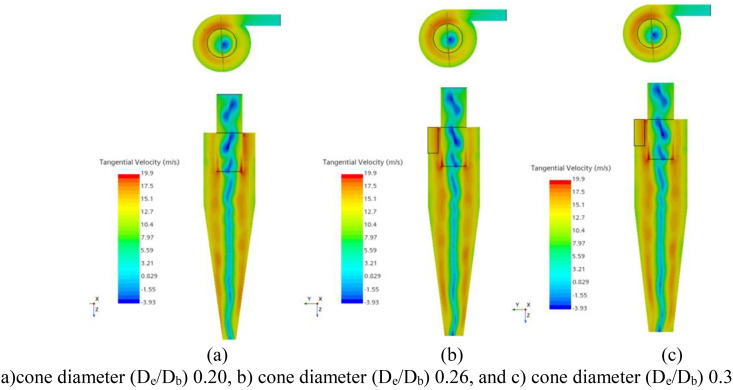
Tangential velocity contours for three bottom cone designs.

The near-wall flow, known as the downward outer flow, contrasts with the upward inner flow at the cyclone’s core. The tangential velocity for the three designs is shown in Fig 15. Cone diameter primarily affects tangential velocity, with a larger diameter generally yielding a higher tangential velocity, as shown in Fig 15(c).

A larger cone diameter can lead to a longer gas flow path, potentially allowing more time for tangential velocity to develop or decay. Fig 15 (a) shows a cyclone with a smaller cone diameter, while Fig 15(c) depicts a cyclone with the largest cone diameter. Fig 15(b) presents the optimized cone diameter of 0.26D

The size of the bottom cone opening in a cyclone directly influences its rotational speed. A wider opening typically results in slower rotation, while a narrower opening lead to faster rotation.

**Fig 16:**
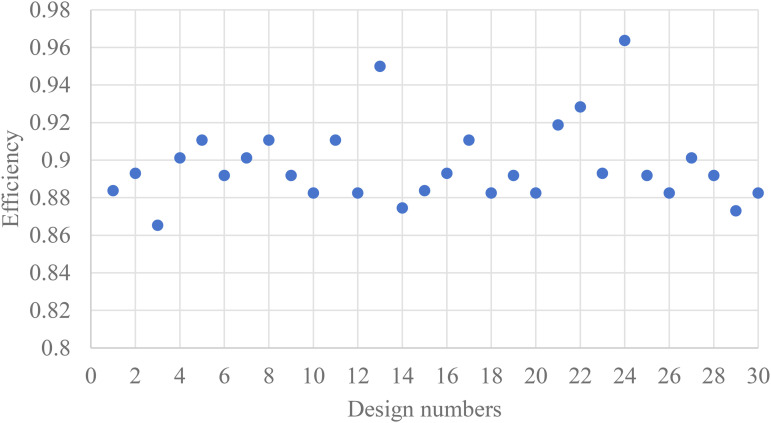
Cyclone efficiency vs designs.

Fig 16 shows the cyclone efficiency for all 30 designs. The optimal cone diameter, determined to be 0.26, is critical for balancing several factors that affect cyclone performance. This diameter strikes a balance between pressure drop, axial velocity, and tangential velocity to maximize separation efficiency. A smaller cone diameter might enhance separation efficiency due to higher centrifugal forces, but at the cost of increased pressure drop and potential particle re-entrainment. A larger cone diameter, while reducing pressure drop and wear, might lower separation efficiency due to reduced centrifugal forces. Thus, determining the optimal cone diameter is crucial for achieving the desired balance between high separation efficiency and manageable pressure drop.

### 4.3 Influence of vortex finder dimension

Optimizing the vortex finder length and diameter is essential for the effective functioning of cyclone separators. The appropriate dimensions of the vortex finder help balance high separation efficiency with an acceptable pressure drop, enhancing the cyclone’s overall performance. Table 7 provides the ranges of inlet height and width used in the experimental design. A total of 30 design experiments (DOE) were conducted.

**Table 7:** Variation of vortex finder parameters.

Sr. No.	Parameters	Range
1	Vortex diameter ratio (**D**_**v**_**/D**_**b**_)	0.25 to 0.75
2	Vortex length ratio (**L**_**v**_**/D**_**b**_)	0.25 to 0.75

**Fig 17:**
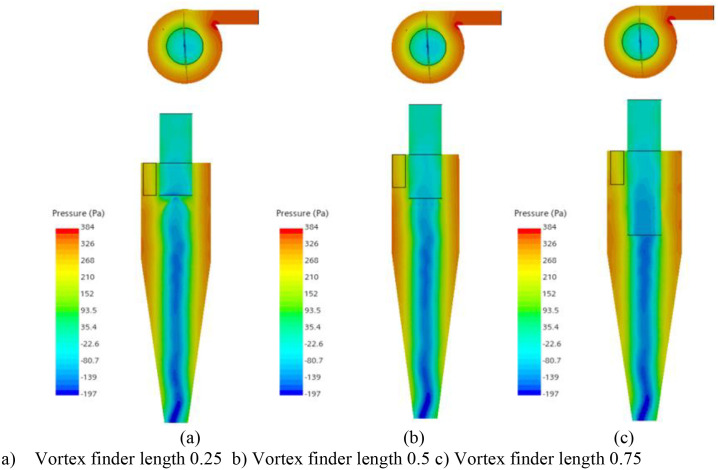
Pressure contours for three vortex finder designs.

Pressure contours for three designs are given in Fig 17. A longer vortex finder can increase the pressure drop within the cyclone shown in Fig 17(c). This is because the extended length increases resistance to the exiting fluid, resulting in greater energy loss. Conversely, a shorter vortex finder is shown in Fig 17(a). reduces the pressure drop, allowing the fluid to exit more freely. However, this might affect the separation process, as it could allow more particles to be carried out with the exiting fluid. In Fig 17(c), a vortex finder length of 0.5D strikes an optimal balance between pressure drop and efficiency, ensuring efficient cyclone operation.

**Fig 18:**
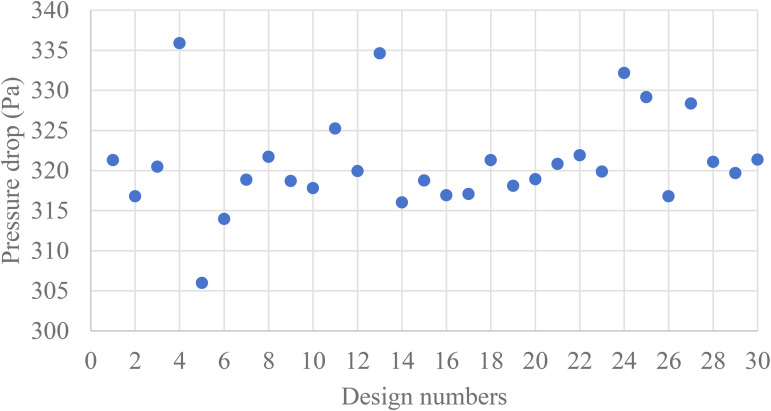
Pressure drops for all designs.

The pressure drop is lowest for design 5 and highest for design 4 across all designs, as shown in Fig 18. Vortex finder length 0.25 and 0.75, respectively, for design 5 and design 4. The size of the vortex finder opening in a cyclone directly influences the pressure drop across it. A wider opening typically results in lower resistance to gas flow, thereby reducing the pressure drop. Conversely, a narrower opening can increase the resistance, leading to a higher pressure drop. The axial velocity is related to the downward and upward flow streams. The axial velocity profiles differ slightly between designs.

**Fig 19:**
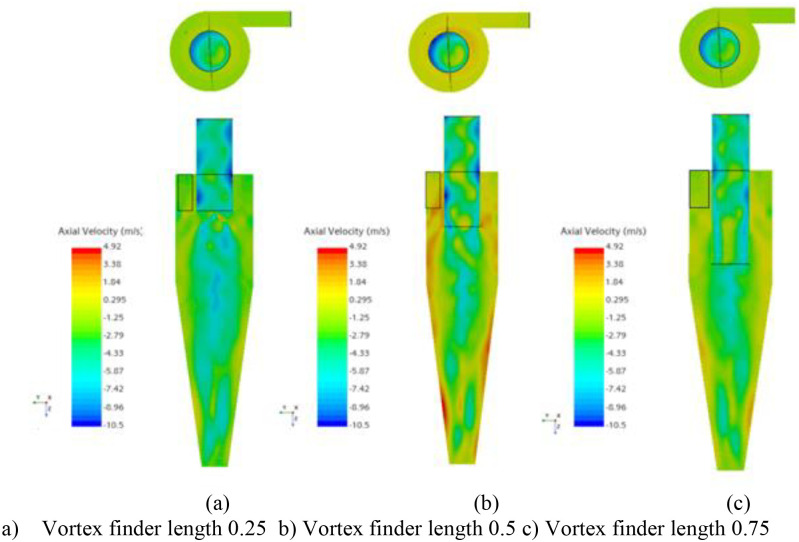
Axial velocity contours for three vortex finder designs.

Extending the vortex finder length typically reduces the axial velocity near the cyclone wall by redirecting the flow path, thereby lowering axial velocity gradients and potentially improving particle residence time. A shorter vortex finder, as shown in Fig 19(a), can accelerate the flow of fluid out of the cyclone, reducing the time particles spend inside. This can reduce separation efficiency, as particles may not have enough time to separate effectively. The contours of axial velocity are shown in Fig 19. A longer vortex finder shown in Fig 19(c) minimizes the short-circuiting of fluid, preventing fluid from bypassing the primary separation zone and exiting the cyclone prematurely. In Fig 19 (c), the vortex finder length of 0.5D achieves an optimal balance between pressure drop and axial velocity within the cyclone.

**Fig 20:**
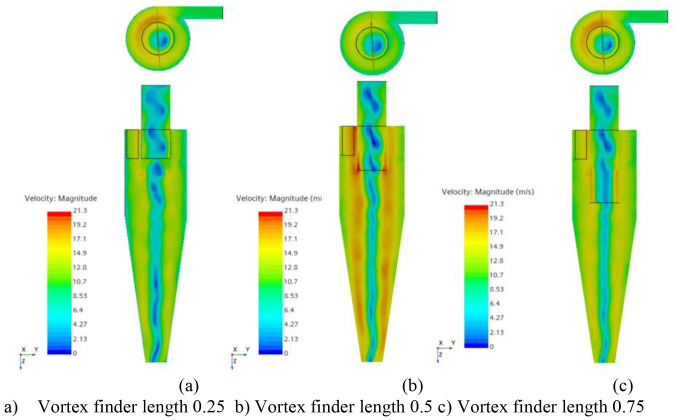
Velocity contours for three vortex finder designs.

**Fig 21:**
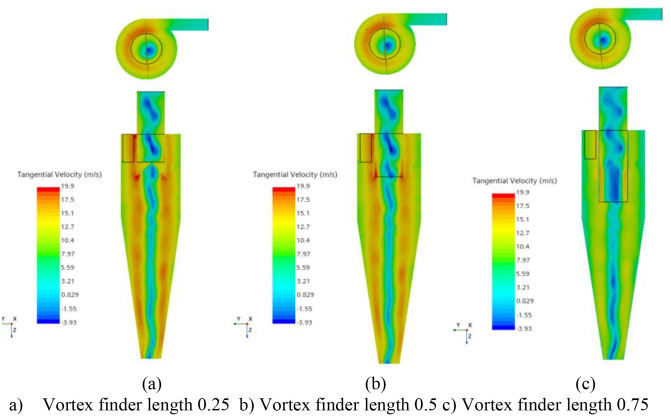
Tangential velocity contours for three Vortex Finder designs.

Fig 20 illustrates the velocity magnitude, which has a minimum impact for all three designs as shown in Fig 20 (a), (b), and (c). Fig 21 shows tangential velocity contours. A longer Vortex Finder affects the tangential velocity distribution by extending the region where centrifugal forces act, potentially improving separation for certain particle sizes while also increasing the pressure drop.

A shorter vortex finder may result in higher tangential velocities near the cyclone outlet, which can enhance the separation of smaller particles but may also increase wear on the cyclone walls. The size of the vortex finder opening in a cyclone directly influences its rotational speed. A wider opening typically results in slower rotation, as shown in Fig 21(c), while a narrower opening lead to faster rotation, as shown in Fig 21(a). In Fig 21 (b), it achieves the optimal balance between pressure drop and tangential velocity, resulting in the cyclone’s peak performance.

**Fig 22:**
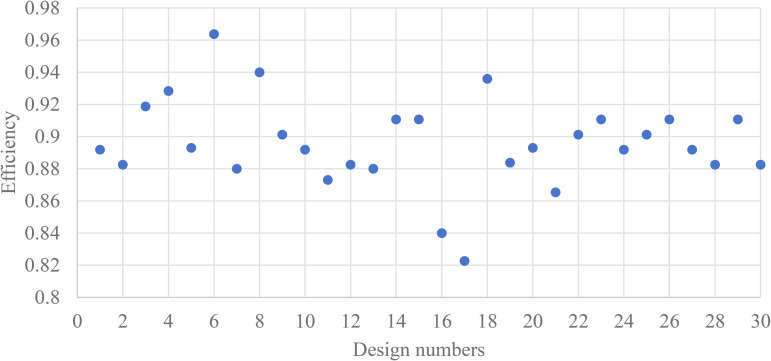
Cyclone efficiency.

Cyclone efficiency for all 30 designs is shown in Fig 22. Cyclone efficiency is lowest for design 17 (vortex length 0.25) and highest for 6 (vortex length 0.66) in all designs. The cyclone’s separation efficiency is directly tied to the size of its vortex finder. A longer vortex finder provides a greater distance for the gas and particles to travel before exiting the cyclone. This increased residence time allows for more opportunities for particles to be separated from the gas stream. An optimal design (vortex length 0.5) is achieved to maximize separation efficiency while managing pressure drop and energy consumption.

### 4.4 Overall optimized geometry

**Fig 23:**
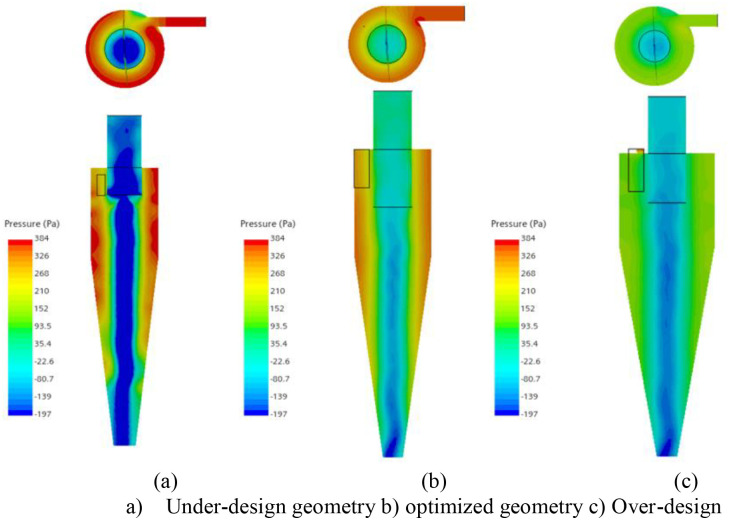
Pressure contours for three designs.

Multi-geometrical parameters are considered in design optimization. The ranges of all six design parameters vary during the DoE, as shown in Table 4. A total of 30 DOEs are performed. Fig 23 shows the pressure contours for three designs. Extremely high pressure is observed in under-design geometry, Fig 23(a), and low pressure is observed in over-design geometry, Fig 23(c). Due to the high swirling velocity, a region of lower pressure (negative-pressure zone) exists in the main area (also known as the forced vortex zone) in all three cases. Fig 23(b) illustrates the optimized geometry, showing that the pressure drop falls within the anticipated range.

**Fig 24:**
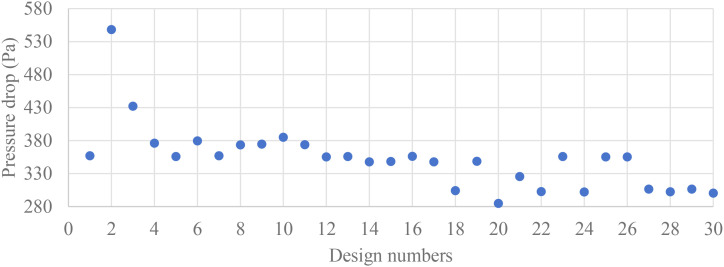
Pressure contours for three designs.

Fig 24 represents the pressure drop for all designs. The highest-pressure drops are for design 2, and the lowest are for design 20.

**Fig 25:**
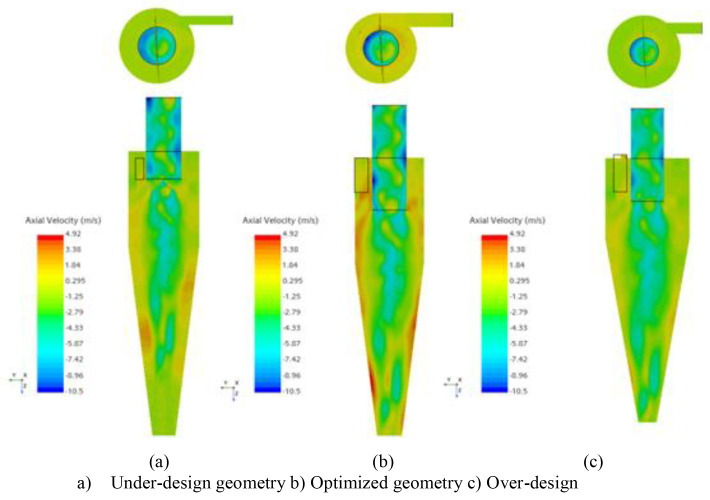
Axial velocity contours for three designs.

The axial velocity is related to the downward and upward flow streams. The axial velocity profiles differ slightly between the three designs, as shown in Fig 25. Fig 25(a) highlights an under-designed cyclone where axial velocity is high compared to the other two designs, while Fig 25(c) exhibits an over-designed cyclone with the minimum axial velocity. In contrast, Fig 25(b) presents the optimized design.

**Fig 26:**
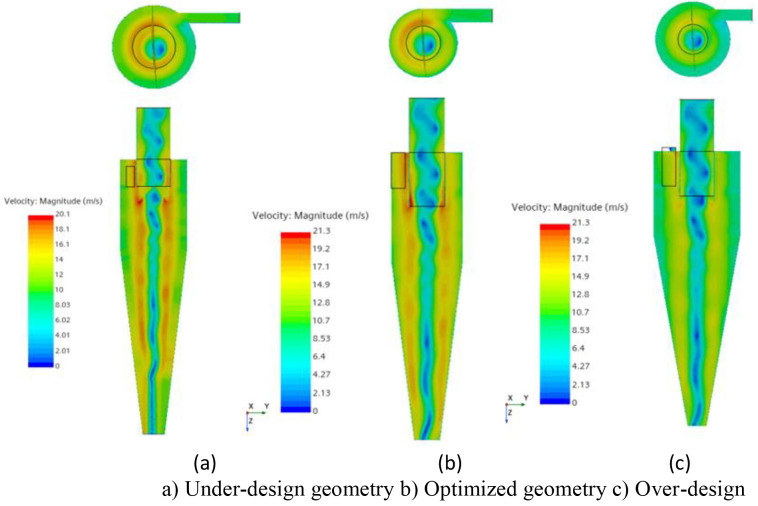
Velocity contours for three designs.

The results indicated minimal acceleration within the cyclone, with only slight variations in velocity magnitude, as shown in Fig 26. Fig 26 (a) represents the under design, and Fig 26(c) is the over design, where the cyclone body diameter is highest. Fig 26(b) illustrates the optimized design.

**Fig 27:**
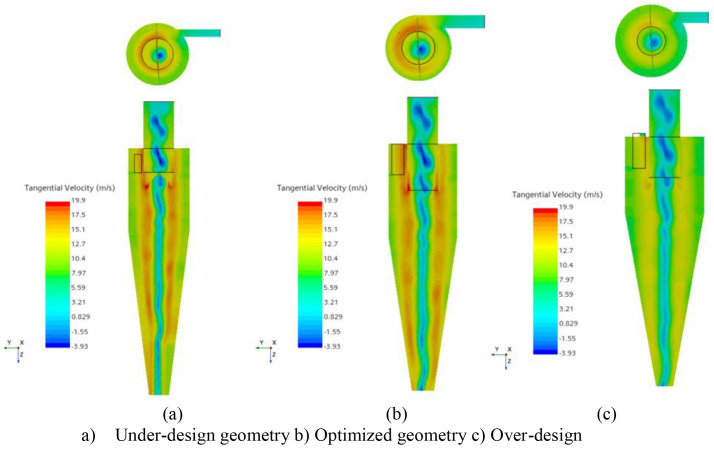
Tangential velocity contours for three designs.

Fig 27 represents the tangential velocity for three designs. It is observed that tangential velocity is lowest with the over-design case, as shown in Fig 27(c), and highest in the under-design geometry, as shown in Fig 27(a). Fig 27(a) depicts the under-designed cyclone, Fig 27 (c) shows the over-designed cyclone with the largest body diameter, and Fig 27(b) illustrates the optimized design.

**Fig 28:**
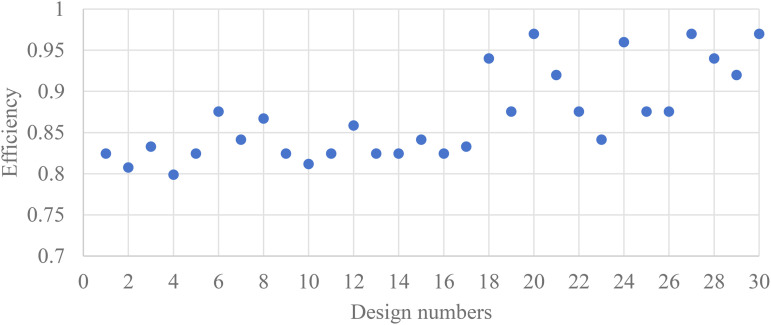
Cyclone efficiency for all designs.

Fig 28 illustrates the cyclone efficiency for all designs. The best efficiency is for design20, and the worst is 0.8 (80%) for design4.

### 4.5 Comparative performance study of the optimized cyclone separator

In addition to the geometric comparison, the performance of the optimized cyclone design was evaluated in terms of pressure drop and internal flow characteristics under identical operating conditions, thereby demonstrating the influence of geometric modifications on separator performance. The Design of Experiments (DoE) approach enables systematic investigation of the effects of key design parameters (e.g., cyclone dimensions and inlet configuration) on performance indicators such as collection efficiency and pressure drop using a limited number of simulation cases. However, the reliability of the DoE-based predictions must be verified. Therefore, the DoE model was validated through comparison with well-established experimental data for the Stairmand cyclone reported by Hoekstra [46], which is widely used as a benchmark case in cyclone CFD studies. This comparison supports the applicability of the adopted modelling framework for cyclone design optimization.

Standard cyclone dimensions have been studied by Kashan [48], who presented the geometric parameters for different types of cyclone separators: high-efficiency, conventional, and high-throughput, as shown in Table 8. The comparison of cyclone dimensions is presented in this table. The geometric parameters of the present optimized cyclone design fall between the categories of high- efficiency and conventional cyclone separators.

**Table 8:** Comparison of cyclone dimensions (geometric parameters).

	Inlet dimensions		Vortex finder		Cone length and particle exit diameter		
Cyclone dimensions ratio (in row)	I_H_/D_b_	I_W_/D_b_	D_v_/D_b_	L_v_/D_b_	L_b_/D_b_	L_c_/D_b_	D_e_/D_b_
High efficiency	0.5	0.2	0.5	0.5	1.5	2.5	0.375
Conventional	0.5	0.25	0.5	0.625	2.0	2.0	0.25
High throughput	0.75	0.375	0.75	0.875	1.5	2.5	0.375
**Optimal design (Present work)**	**0.5**	**0.21**	**0.5**	**0.55**	**1.5**	**2.31**	**0.26**

To assess the significance of the predicted reduction in pressure drop in the case of the optimized cyclone (present work), the high-efficiency, conventional, high-throughput, and optimized cyclone geometries were simulated under identical numerical conditions, including mesh structure, boundary conditions, turbulence model, and convergence criteria. It was revealed that the simulated pressure drop (present optimized design) was 285 Pa, and the cyclone efficiency of 96%. The cyclone efficiency (98%) in the case of a high efficiency type cyclone is higher than that of the present optimized design, despite the increment in the pressure drop (430 Pa) to achieve higher particle separation. The high-throughput cyclone has wider inlet dimensions and handles high gas volumes. Therefore, the cyclone efficiency of the high-throughput type cyclone is far lower than that of the present optimized design. Table 9 indicates that the optimized design provides a measurable improvement in hydraulic performance, meaning higher cyclone efficiency with a considerable pressure drop. The present optimized design (design 20) of a cyclone separator effectively balances pressure drop and efficiency, indicating a well-performing cyclone design. This supports the conclusion that the optimized design can contribute to long-term energy savings.

**Table 9:** Comparison of cyclone separator performance.

Type of cyclone	Pressure drop (Pa)	Cyclone efficiency (%)
High efficiency	430	98
Conventional	310	88
High throughput	320	66
**Optimal design** **(Present work)**	**285**	**96**

## 5 Conclusions

This work represents the use of computational analysis to optimize cyclone separator designs and to verify the accuracy of the proposed model. The optimized design is validated against existing experimental data. This validation process confirmed the predictive power of our models for airflow patterns and overall cyclone separator efficiency.

The conclusions drawn in this study are based on the present CFD model configuration, computational settings, and associated modelling assumptions.

1. The optimal ratio of inlet width (I_w_) to inlet height (I_H_) was found to be 0.42, which effectively balances pressure drop and separation efficiency.
2. This analysis identified a strong correlation between a decrease in cone bottom size and a significant increase in collection efficiency. Variations in cone size won’t significantly impact pressure drop.
3. The optimized geometry features an inlet height ratio of 0.5, an inlet width ratio of 0.21, a particle exit diameter ratio of 0.26, a bottom cone length of 2.31, and vortex finder dimensions of 0.5 in diameter and 0.55 in length.
4. The optimized geometry configuration results in a pressure drop of 285 Pa and an efficiency of 96%, positioning it between conventional and high-efficiency cyclone models.
5. DoE offers a structured and efficient approach to exploring the influence of multiple design variables (geometrical parameters) on performance metrics (collection efficiency, pressure drop) within a limited number of experiments.
